# Monthly Memoranda

**Published:** 1854-02

**Authors:** 


### EDITORIAL DEPARTMENT.

MONTHLY MEMORANDA.

*Epidemic Erysipelas.*—The selected article on this subject is worthy the considerate attention of the reader. So far as we have been able to trace the history of this disease, it does not altogether accord with the experience of the author, with regard to its malignancy and fatality. It has appeared in different localities in the Western States, and such has been its fatality in some of these visitations, that the populace were in almost as much dread of it as of the cholera itself.

The author is doubtles right in avowing the disease to be an eruptive zymotic. The symptoms show extensive blood poisoning, and that it originates in some peculiar atmospheric constitution; and from the fact that it has a moie malignant aspect in certain miasmatic districts, it may find aggravations from this source, either from its predisposnent influences or from complications. It is often accompanied in the same locality with other exanthemata, as scarlatina, varicella. and sometimes ruibeola; and the simultaneous prevalence of puerperal peritonitis has been so often observed as to be looked for with reasonable certainty.

For the last two months, or more, it has prevailed in a locality in this county, and in adjoining districts of other counties, constituting an area of some ten miles square. Within this limit it has prevailed more or less during the above period. Compared with some other visitations, it has been mild, a few fatal cases only having occurred. In one family five cases occurred, almost simultaneously, three of which were severe attacks, and in one instance it proved fatal. In its further spread other fatal cases occurred. A valuable medical friend, Dr. Atkinson, residing in that locality, contracted the disease, in return for his devotion, labors, and skill in behalf of the afflicted, and although brought down very low, the last intelligence from him was favorable to his recovery. Mingling with this disease, there have been a number of well marked cases of scarlatina, and a few of varicella. The disease was ushered in by rigors and inflammation of the throat, which were soon followed by fever, pain in the head, and these if not arrested passed rapidly into the typhoid form. As we have the promise of a full report of the history of this epidemic from those entirely competent to the task, we forbear any further remarks at this time, save only that as soon as received, they will be promptly given to our readers.

*Poisoning with Opium.*—Dr. Nye of Lynn, Mass., suggests in the N. H. Medical Journal, a method of inducing vomiting in those difficult cases of poisoning with opium, where the narcotic induces such such profound insensibility of the stomach, that that organ will not respond to the usual stimulus of an emetic. The patient, "previously in good health, while in a passion, induced by some domestic feud, procured and swallowed from ʒv to ʒviij of tinct. opii. In a short time she became quite stupid. The poison had been swallowed forty five minutes, when I arrived, and she had taken large draughts of tepid water, and mustard.

She was quite stupid; cerebral vessels distended; face flushed, and could walk only with the assistance of two persons. Wishes to be left alone, that she may go to sleep. While held erect, her head falls to one side, and she breathes with some difficulty.

Ordered an emetic of pul. ipecac and pul. sinapis, aa. ʒij. in tepid water, to be followed by several tumblers of water, the head to be showered with cold water. No vomiting occurred in thirty minutes. Repeated the emetic. Introduced a feather into the œsophagus. Waited fifteen minutes; no relief; symptoms more grave. Ordered two tumblers of diluted vinegar, to be immediately followed by ʒij. of carb. potassa, in water. A powerful efferevscence took place, which instantly produced most copious vomiting.

It is suggested that the above process may in most cases answer instead of the stomach pump—in those cases, however, where from insensibility or obstinacy, the patient refuses to swallow, the stomach pump may be introduced, and the solution of any carbonated alkali and a corresponding acid injected, when equally favorable results ensue. A physician cannot have too many reliable expedients to fall back upon, in such emergencies.

*Cholera.*—The Medical Examiner states that the foreign journals convey the welcome intelligence "that the cholera is on the decline throughtout all Europe. An excellent regulation prevails in many parts of England, having for its object the checking of the spread of this disease. We allude to the appointment of physicians as sanitary Inspectors of their several districts. Their duties are to report nuisances of all kinds to the local boards of health and to visit, in case of death, the house in which it took place, and administer prompt medical relief to every case of diarrhœa. The latter provision for the poor, who are often both negligent and apathetic, is considered necessary, as there is a very general belief that this premonitory condition exists in every instance.

Another interesting fact seems to have been established; which is, that the same localities, even the same houses have been visited by this disease, in its several epidemics.

We mention these matters, as they have an important bearing upon the direction, which should be given to our preventive and sanitary efforts, should we again have the misfortune to be visited by this pestilence.

*Local Etherization.*—It seems that the *vapor* of ether, applied to sensitive surfaces, is capable of affording speedy relief from severe pain. We have seen a number of cases involving this principle, and it is capable undoubtedly of a wide application. A French Medical work states that it has been very efficacious in relieving a large number of cases of neuralgia of the ear, and also in curing those cases of erythism in which the chief symptoms consists in distressing humming and ringing in the ears. This is local etherization. A few drachms of ether are placed in a bottle, the mouth of which is adapted to the external meatus. The bottle is grasped in the palm of the hand, and the animal warmth suffices to votalize the ether. Dr. Delioux states that the effects of this treatment are rapid and permanent.

*Iodide of Zinc.*—The Medical Times and Gazette states that during the last six months an extended series of trials of a new drug, the iodide of zinc, has been made in Guy's Hospital, on patients under the charge of Dr. Barlow. The salt has been prepared, in the form of a syrup, of which the dose is the same as of iodide of iron. Dr. Barlow considers it especially indicated in cases in which the zinc alone might be too stimulating, and in those in which it is desirable to have the effects of both the ingredients. Chorea, struma, cachexia, and some forms of hysteria, from the chief in which it has been tried. In one case of severe strumous superficial lugus, it has been continued for many months and in full doses, with great apparent benefit. It is doubtful whether the cases of chorea have improved more rapidly than might have been expected to do under the usual treatment by the sulphate.

*Fractures.*—We saw, some years since, in an eastern Hospital, of great celebrity, fracture of the femur treated in a primitive manner:

the limb was drawn down by a couple of bricks, hanging by a string over the foot board of the bed, and connected with a bandage upon the foot of the patient. Prof. Judkins, writing from Paris to the Ohio Med. Journal says, M. Jobert treats fractures without the aid of splints; in some cases without bandages. In one case, bands for extension and counter-extension, were all the dressings that were placed about the limbs. I will give you the result after I have ascertained it myself.

By the way, a case managed in '53, now in the wards of Hospital Cochin, is being treated for fracture of the neck of the femur—intra capsular. The only aparatus made use of, is a simple cross-piece at the foot to keep the foot steady, and prevent any lateral movement or rolling motion of the leg. There is no extension or counter-extension used, and no dressing whatever near the region of fracture.

This seems like the very ultimatum of simplicity. But, when we regard the complexity of encumbering applications with which surgery is sometimes overloaded, an excess of simplicity may be almost justifiable, and at least useful in recalling us from the opposite ultraism, to a healthful and reasonable medium.

